# Challenges in the management of acute lithiasic cholangitis due to a long-retained plastic biliary stent: A case report

**DOI:** 10.1016/j.ijscr.2024.109690

**Published:** 2024-04-23

**Authors:** Mohamed Ali Chaouch, Ahmed Hadj Taieb, Aymen Kawach, Hanen Zenati, Besma Gafsi, Faouzi Noomen

**Affiliations:** a Department of Visceral and Digestive Surgery, Monastir University Hospital, Monastir, Tunisia; b Department of General Surgery, Sidi Bouzid Regional Hospital, Sidi Bouzid, Tunisia; c Department of Anesthesia, Monastir University Hospital, Monastir, Tunisia

**Keywords:** Acute cholangitis, Biliary stent, Choledocholithiasis, Endoscopic sphincterotomy, Surgical intervention, Case report

## Abstract

**Background and importance:**

This case report focuses on a rare cause of acute lithiasis cholangitis, which is residual choledocholithiasis on a plastic biliary stent that was placed nine years prior.

**Case presentation:**

An 87-year-old male, with a history of hypertension and previous surgery for gallstone disease including cholecystectomy and placement of a Kehr drain in 2006, was diagnosed with residual stones in 2008 and received a plastic biliary stent after endoscopic sphincterotomy. Lost to follow-up for nine years, he presented with acute lithiasis cholangitis characterized by fever, conjunctival jaundice, leukocytosis, CRP elevation, and biochemical signs of cholestasis. CT imaging revealed choledocholithiasis on the biliary stent. The patient underwent surgical intervention, during which a dilated bile duct was discovered, a complete tangential choledocotomy was performed, and the stent/stone complex along with additional choledocholithiasis was removed. A choledochoduodenal anastomosis was subsequently performed.

**Discussion:**

The use of plastic biliary stents can paradoxically lead to the formation of biliary stones, a condition termed “stentolith”. Such scenarios emphasize the complications arising from prolonged stent presence, including bacterial proliferation and the consequent formation of calcium bilirubin stones. While endoscopic removal of these stent-stone complexes has been successful in a few cases, surgical intervention is often required due to the risks associated with endoscopic extraction, such as potential duodenal perforation. The choice of lithotripsy technique for endotherapy depends on availability and patient-specific factors.

**Conclusion:**

This complication highlights the importance of patient education, meticulous record-keeping, and regular follow-up to prevent such outcomes.

## Highlights

• This case report underscores the rare complication of acute lithiasic cholangitis arising from a plastic biliary stent retained for nine years, leading to choledocholithiasis.
• The successful surgical intervention, including choledocotomy and choledochoduodenal anastomosis, following the failure of endoscopic management due to prolonged stent retention, emphasizes the need for a tailored approach in complex cases.
• The report highlights the importance of patient follow-up and education regarding the limited lifespan of plastic biliary stents to prevent severe complications such as the formation of a stent-stone complex.

## 1 Background and importance

Acute lithiasic cholangitis is a common medical and surgical emergency. Interventional endoscopy allows both acute management and can serve as definitive treatment [1,2]. The management of forgotten long-term biliary stents, as highlighted in a study, often involves endoscopic interventions to address complications like acute cholangitis associated with CBD stones (Complications and management of forgotten long-term biliary stents). However, surgery, particularly biliary-enteric anastomosis, is indicated in cases where the appropriate technical resources are unavailable or when inherent difficulties arise with the technique itself. This case report, reported according to SCARE guidelines [3], aimed to highlight a rare aetiology of acute lithiasis cholangitis, namely residual choledocholithiasis on a plastic biliary stent placed nine years ago.

## 2 Case presentation

**Fig. 1:**
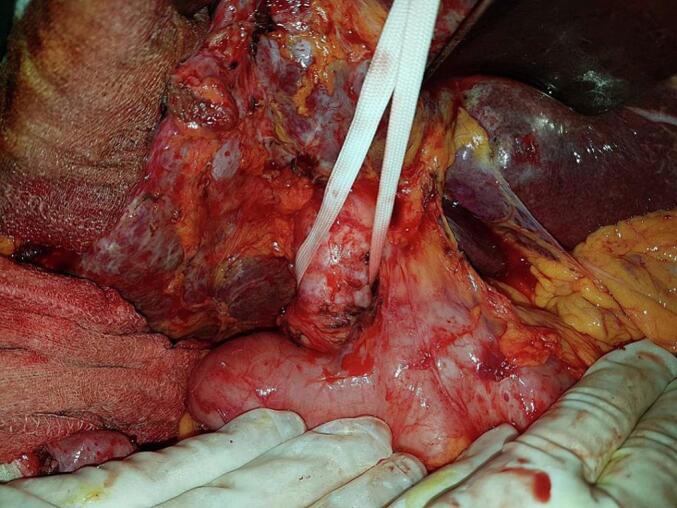
Dilated bile duct.

**Fig. 2:**
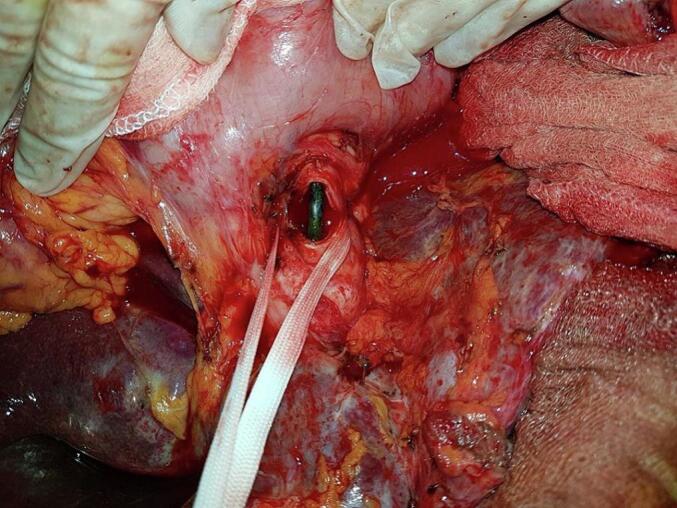
Biliary stent in the bile duct after the choledocotomy.

**Fig. 3:**
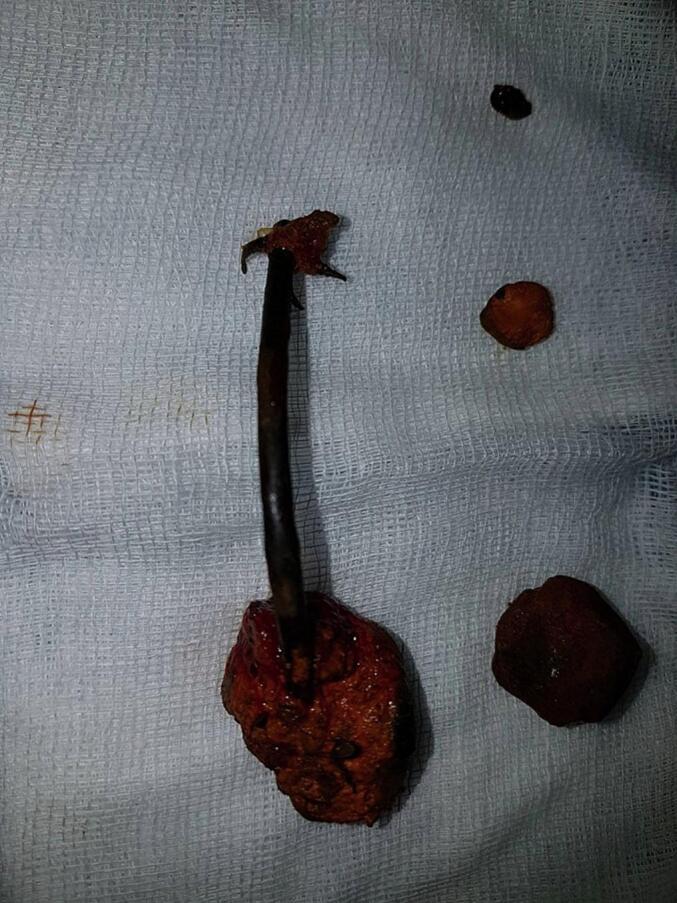
The stent/stone complex with three other choledocholithiasis extracted from the bile duct.

**Fig. 4:**
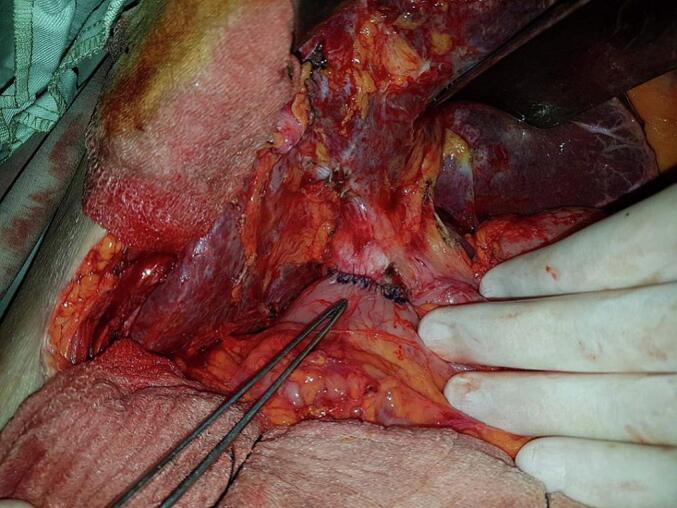
Choledochoduodenal anastomosis.

An 87-year-old man, hypertensive and treated with conversion enzyme inhibitors, underwent surgery in 2006 for gallstone disease associated with common bile duct stones. Through a right subcostal approach, he underwent cholecystectomy with the placement of a Kehr drain. Immediate postoperative recovery was uneventful. Two years after the procedure, the diagnosis of residual stones was made. The patient underwent endoscopic sphincterotomy with placement of a plastic biliary stent in 2008. He was lost to follow-up for nine years, then presented nine years later with acute lithiasic cholangitis. On examination, the patient was febrile at 38 °C with conjunctival jaundice, the blood pressure was 90/50 mmHg, the breathing rate of 28 per minute, and the heart rate of 120 per minute. Laboratory findings revealed leukocytosis at 13,000 cells/mm^3^, CRP = 63 mg/l, and evidence of biochemical cholestasis with hyperbilirubin of 180 IU/ml. An abdominal computed tomography scan (CT scan) showed choledocholithiasis on the biliary stent. The patient was started on antibiotics (Piperacillin/Tazobactam: 3.375 g IV every 6 h) and underwent surgery via a right subcostal approach after an open coelioscopy failure to create pneumoperitoneum due to huge adhesions. In the exploration, we found a highly dilated bile duct (Fig. 1). We decided to perform a complete tangential choledocotomy, which allowed us to extract the stent/stone complex with three other choledocholithiasis (Fig. 2, Fig. 3). A choledochoduodenal anastomosis was performed (Fig. 4) because we have some concerns about incomplete clearance with the existence of distal obstruction. The postoperative course was uneventful.

## 3 Discussion

This case underscores the critical importance of interdisciplinary collaboration in the management of complex biliary diseases. The convergence of gastroenterology, radiology, and surgery was pivotal in achieving a favorable outcome for our patient, highlighting the value of a multi-disciplinary approach in navigating complicated clinical scenarios.

Biliary stents have a limited lifespan, typically around three months for plastic biliary stents, due to lumen occlusion. Several papers have addressed this issue [4]. These plastic stents if kept for a prolonged period promote bacterial proliferation, and release of bacterial beta-glucuronidase, which results in the precipitation of calcium bilirubin. Calcium bilirubin is then aggregated into stones by an anionic glycoprotein. Thus, the stents themselves end up causing the primary disorder for which they were inserted in the first place [5]. The de novo formation of biliary stones around the stent was reported in a few case reports. These may lead to a stone-stent complex assuming a lollipop, dumbbell, or the stent shape. Bansal and his colleagues were the first to term this complex ‘stentolith’ in 2009 [6]. Those patients with forgotten stents commonly present with abdominal pains, obstructive jaundice, and cholangitis. They usually have deranged liver function tests and dilated biliary tracts on abdominal ultrasound [7]. Our experience adds to the growing body of literature advocating for heightened vigilance and proactive management strategies for patients with biliary stents. The development of a stent-stone complex, as observed in this case, serves as a stark reminder of the potential for severe complications arising from delayed stent removal.

The management of those cases needs to be tailored to each case after careful history and investigations. Although there are few reports of successful endoscopic clearance of stentoliths, the majority of the patients require surgical intervention [8,9]. The excessive force in an attempt to remove endoscopically the stent along with the scope, even after a complete sphincterotomy, could lead to duodenal perforation [9]. Endotherapy could use mechanical lithotripsy, extracorporeal shock wave lithotripsy (ESWL), electrohydraulic lithotripsy (EHL) or laser lithotripsy as an effective method in fragmenting large stones [10]. The method of choice depends on availability and is individualized accordingly [11,12].Thus, patients who receive plastic stents should be warned about this complication and be under close observation. Conversely, physicians encountering patients with ageing plastic stents in their common bile duct should promptly refer them for endoscopic removal and clearance of bile debris to prevent the occurrence of severe complications [9,13]. The successful surgical intervention in our patient also illustrates the evolution of surgical techniques and the critical role they play in managing complications that were once deemed insurmountable. It reinforces the notion that surgical innovation continues to be an indispensable asset in the armamentarium against complex biliary pathology.

## 4 Conclusion

Our case not only underscores the clinical challenges associated with retained biliary stents but also emphasizes the broader implications for healthcare systems. It calls for a concerted effort to improve patient tracking, follow-up care, and interdisciplinary communication to prevent such occurrences, which can significantly impact patient outcomes and healthcare resources.

## Consent

Written informed consent was obtained from the patient for publication of this case report and accompanying images. A copy of the written consent is available for review by the Editor-in-Chief of this journal on request.

## Ethical approval

Not applicable.

## Funding

This research received no specific grant from the public, commercial, or not-for-profit sectors.

## Author contribution

All the authors participate in the treatment of the patients, writing, and approved the manuscript.

## Guarantor

Mohamed Ali Chaouch.

## Research registration number

N/A.

## Conflict of interest statement

No conflict of interest to disclose.
